# Bayesian spatiotemporal evaluation of bovine anaplasmosis seroprevalence in Missouri (2010–2021)

**DOI:** 10.3389/fvets.2025.1658248

**Published:** 2026-01-23

**Authors:** Ram K. Raghavan, Rosalie Ierardi, Frank Badu Osei, Shuping Zhang

**Affiliations:** 1 Department of Pathobiology and Integrative Biomedical Sciences, College of Veterinary Medicine, University of Missouri, Columbia, MO, United States; 2 Department of Public Health, College of Health Sciences, University of Missouri, Columbia, MO, United States; 3 MU Institute of Data Science and Informatics, University of Missouri, Columbia, MO, United States; 4 Veterinary Medical Diagnostic Laboratory, College of Veterinary Medicine, University of Missouri, Columbia, MO, United States; 5 Department of Natural Resources, Faculty of Geo-Information Science and Earth Observation, University of Twente, Enschede, Netherlands

**Keywords:** *Anaplasma marginale*, Bayesian spatiotemporal analysis, bovine anaplasmosis, imputation model, missingness

## Abstract

Bovine anaplasmosis, caused by the rickettsia *Anaplasma marginale*, is an economically important and globally distributed tick- and blood-borne disease of cattle. Although cases are known to be widespread in Missouri, current spatiotemporal trends, presence of high-risk areas, and any potential drivers for disease trends in Missouri are poorly documented. To address these knowledge gaps, this study analyzed spatiotemporal patterns of annual, county-level anaplasmosis case counts using a Bayesian hierarchical framework. Seropositive cases of anaplasmosis detected at the University of Missouri Veterinary Medical Diagnostic Laboratory (*n* = 1,944) between the years 2010–2021 were used to construct data-driven Bayesian hierarchical models. All the models consisted of imputation sub-models to alleviate issues related to missing observations from spatiotemporal units (114 counties and 1 independent city, 12 years). Three progressively complex models with different assumptions for capturing the spatial, temporal, and spatiotemporal interactions that explained the variability in case counts were prepared. Model-1 included linear predictors decomposed into structured and unstructured terms for the temporal and spatial processes. Model-2 included separate temporal terms for smoothing each spatial entity and spatial smoothing terms for each temporal entity. This model was extended in Model-3, which included space-time interaction effect using first-order conditional autoregressive (CAR) priors. Based on the Deviance Information Criterion (DIC), Model 3 was superior at explaining space/time variability in the detected seropositive cases of bovine anaplasmosis. These findings indicate that distribution and risk of bovine anaplasmosis seroprevalence in Missouri are non-uniform, and potentially driven by environmental and/or management factors, operating at local and regional scales, that when identified could inform mitigation strategies.

## 1 Introduction

Bovine anaplasmosis, caused by the rickettsia *Anaplasma marginale*, is an economically important tick- and blood-borne disease of cattle found worldwide. Its clinical effects of severe anemia, weight loss, spontaneous abortions, and death negatively impact cattle welfare and create a significant economic burden for cattle producers (1). A vaccine that is consistently safe and effective has proved elusive (2). Treatment and control usually depends on administration of oral and/or injectable tetracyclines; however, this approach is not always effective (3) and may be associated with the development of antimicrobial resistance (4). Cattle that survive acute infection are lifelong subclinical carriers of *A. marginale* and represent a key reservoir of infection for other cattle through tick transmission, inadvertent mechanical transmission via blood-contaminated needles (5) and veterinary instruments, and mechanical transmission via certain biting flies (6, 7).

Evidence suggests that bovine anaplasmosis is becoming more prevalent and more geographically widespread (8–13). Spatiotemporal modeling demonstrated increases of anaplasmosis in Kansas since 2005 (14), where over 50% of beef herds are infected (15). Human tick-borne illnesses follow a similar trend, having increased significantly over the last decade (16), particularly in the midwestern U.S. Concurrently, expansion of the geographic distributions and active seasons of medically important ticks in North America has been well documented (17–20), while human encroachment on tick habitats increases the likelihood of encounters (21).

In the U.S., bovine anaplasmosis is vectored by *Dermacentor* spp. including the American dog tick (*D. variabilis*), the Rocky Mountain wood tick (*D. andersoni*), the winter tick (*D. albipictus*), and the western dog tick, *D*. *occidentalis* (22). Both *D. variabilis* and *D. albipictus* are established in Missouri, although *D. albipictus* preferentially feeds on cervids (23). Distribution of tick-borne diseases, such as bovine anaplasmosis are often concordant with the spatial distribution of their transmitting vectors (17, 24, 25), and are therefore indirectly affected by the natural fluctuations in climate and habitat availability for ticks and their hosts. In the case of bovine anaplasmosis, additional factors that may affect spatiotemporal distribution include management practices (5, 26) and cattle movements leading to comingling of naïve and carrier animals.

Spatiotemporal disease mapping models are a useful tool to describe such disease patterns, more specifically to identify the presence of any clusters of incidences over space, time, or both (27). The presence of such clusters often points to underlying management, environmental, or other factors that could be potentially driving the disease incidence, which can then be targeted for interventions, and resource allocation (28, 29). Making inferences based on crude values of disease incidence over geographic space and time is problematic as the underlying spatiotemporal structure of the data is unaccounted for (27, 30). Bayesian spatiotemporal models provide a robust and flexible platform for space-time analysis of disease incidences and have been utilized to explore the potential effects abiotic covariates such as climate and land cover features in the disease ecology. Such models have been applied to the study of bovine anaplasmosis and other tick-borne diseases (14, 24, 25).

The objective of this study was to evaluate the spatial, temporal, spatiotemporal risk patterns of bovine anaplasmosis in the Midwestern state of Missouri using a Bayesian approach with counts of bovine serum samples that tested positive via competitive ELISA (cELISA) at the University of Missouri Veterinary Medical Diagnostic Laboratory (MU VMDL) between the years 2010–2021.

## 2 Materials and methods

### 2.1 Disease data

The VMDL's laboratory information management system, VetView (University of Georgia, Athens USA), was searched using the “Query Builder” tool for all accessions received January 1, 2010 through December 31, 2021 which included the test code for *A. marginale* competitive ELISA (cELISA). The resulting list of accessions was exported directly from VetView into Microsoft Excel as a .csv file, which included accession number, animal identification, and date of receipt. All case records were reviewed by a single observer (R. Ierardi) and the following items were manually entered into Microsoft Excel: owner's state, county, and city; veterinarian's state, county, and city; animal's age; animal's sex; and, the result of the *A. marginale* cELISA test (1 = positive, 0 = negative). The VMDL's cutoff for a positive result throughout the study period was ≥30% inhibition, according to the manufacturer's instructions (*Anaplasma* Antibody Test Kit, cELISA v2; VMRD, Pullman, WA). The data was curated by excluding cases that met specific criteria (e.g., non-tested samples, samples of inappropriate type, etc.), submitted by owners out-of-state, and submitted by veterinarians out-of-state (Table 1).

**Table 1:** Year-specific and total records of bovine sera, regardless of cELISA result, exported from the VetView laboratory information management system (LIMS) at the University of Missouri Veterinary Medical Diagnostic Laboratory, 2010–2021.

**Year**	**Version 1 (Raw)**	**Non-tested**	**Non-bovine**	**Unsuitable**	**Research**	**Duplicate**	**Version 2 (after criteria exclusions)**	**Version 3 (after removing out-of-state owners)**	**Version 4 (Final, after removing out-of-state veterinarians)**
2010	488	30	10	0	0	0	448	429	429
2011	519	10	3	1	0	0	505	355	310
2012	419	8	9	0	0	0	402	382	381
2013	290	4	5	0	0	0	281	261	256
2014	398	12	7	1	0	0	378	287	287
2015	459	6	16	2	0	0	435	381	381
2016	653	24	15	0	0	4	610	533	530
2017	588	7	2	0	0	2	577	496	496
2018	520	14	1	0	0	0	505	330	330
2019	567	14	4	4	0	0	545	319	319
2020	631	65	0	0	7	2	557	290	289
2021	537	8	0	0	0	0	529	309	308
Total	6,069	202	72	8	7	8	5,772	4,372	4,316

Version 1 = raw data exports; Version 2 = after removing records meeting specific criteria (non-tested, non-bovine, unsuitable sample, research, duplicates); Version 3 = after removing out-of-state owners; Version 4 = after removing out-of-state veterinarians (final dataset). Exclusions between Versions 1 and 2 are detailed by category.

For each of the 12 years, VetView was queried for samples received January 1 through December 31. A species filter was not applied at the time of the original queries for 2010–2019, and non-bovine samples were removed after data entry was completed. A “Bovine” species filter was applied at the time of the original queries for 2020–2021. For each year, VetView would export a list of accessions as a .csv file, each having a column with the date of receipt and another column with a list of comma-separated sample IDs for each accession. Note that VetView's list of samples for a given accession may include multiple sample types destined for different tests. The sample ID column was split by comma delimiter to rows, thus yielding the total number of individual samples received for each year.

### 2.2 Data curation

The queried data Version 1 for 2010–2021 contained 6,069 records, which were subsequently reduced to a subset (Version 2) following the removal of sample IDs that were not tested for anaplasmosis, sample IDs that were not from cattle (usually from other ruminants), and other criteria (Table 1). This resulted in the removal of 297 records. Version 2 (5,772 records) was further filtered to retain only records in which the owner's state was listed as Missouri, or the owner's state was not specified, and the veterinarian's state was Missouri, i.e., if the owner's state was not specified and the veterinarian's state was not Missouri, those records were removed. This resulted in the removal of 1,400 out-of-state records for a new total of 4,372 records (Version 3). Finally, observations where the owner's state was listed as Missouri but the veterinarian was from another state (56 records) were removed; in all of these cases, the owner's residence was in a county adjacent to the state line and often in a metropolitan area—particularly St. Louis—and therefore it was doubtful whether the owner's address was a reasonable proxy for the location of the cattle herd. This resulted in a final total of 4,316 records (Version 4) for analysis.

### 2.3 Geographic data

A 2010 TIGER/Line^®^ shapefile for Missouri counties was downloaded from U.S. Census Bureau (https://www.census.gov/geographies/mapping-files/time-series/geo/tiger-line-file.html). The shapefile included two polygons labeled “St. Louis”; St. Louis County and the independent city of St. Louis, which needed to be distinguished as separate polygons for our analysis. Therefore in RStudio, unique numeric identifiers were assigned to a list of Missouri's alphabetically sorted counties. These numeric county IDs were then appended to the shapefile's attribute table, carefully verifying the county names, and subsequently used as the join field to link with the anaplasmosis dataset using the “Add Join” tool.

### 2.4 Statistical modeling

A binomial hierarchical mixture model was considered for two reasons. Firstly, the disaggregation of the observed data into a space-time framework leads to many missing data points for both the observed outcomes and the expected seropositivity to bovine anaplasmosis in the population. Secondly, this approach seeks to accommodate imperfect detection of anaplasmosis in the population. Hence, the anaplasmosis counts in the present study are modeled as Binomial random variables. This class of models has proved useful in species population distribution models for estimating uncertainties in local abundance and detection probability (31–33). A joint Bayesian analysis and imputation model was developed with a joint density described as


$$\begin{array}{r} f\left(y_{i},n_{i}^{*},n_{i}^{0},m_{i}|\beta \;,\tau_{y\left(•\right)},\tau_{n\left(•\right)},\alpha \right)=f\left(y_{i},n_{i}^{*},n_{i}^{0}|\beta \tau_{y\left(•\right)}\right)\\ \times f\left(n_{i}^{*},n_{i}^{0},m_{i}|\tau_{n\left(•\right)},\alpha \right) \end{array}$$

where the Binomial (positive) outcomes *y*_*i*_ from the samples $n_{i}=\left\{n_{i}^{*},n_{i}^{0}\right\}$ have known $n_{i}^{*}$ and missing $n_{i}^{0}$ sample sizes. The Bayesian *analysis model* is therefore expressed as:


$$\begin{array}{l} f\left(y_{i},n_{i}^{*},n_{i}^{0}|\beta_{0}\tau_{y\left(•\right)}\right)\sim Bin\left(\pi_{i},n_{i}\right)\\ \;n_{i}=\left\{n_{i}^{*},n_{i}^{0}\right\}\\ \;\pi_{i}=S\left(\eta_{i}\right)=\frac{\exp\left(\eta_{i}\right)}{1+\exp\left(\eta_{i}\right)}\\ \;\eta_{i}=\beta_{0}+p_{\gamma \left[j\right]}\gamma_{i\left[j\right]}+\left(1-p_{\gamma \left[j\right]}\right)\delta_{i\left[j\right]}+p_{u\left[k\right]}u_{i\left[k\right]}\\ \;+\left(1-p_{u\left[k\right]}\right)v_{i\left[k\right]} \end{array}$$

where the *i* = 1, …, *N* observations are disaggregated by *j* = 1, …, *J* time steps (years) and *k* = 1, …, *K* spatial locations, in this case, individual counties in Missouri. We project the infection probability π_*i*_on the linear predictor using the logistic sigmoid function $S\left(\eta_{i}\right)$ to ensure an output range between 0 and 1. The coefficient, if exponentiated exp(β_0_), represents the overall odds of anaplasmosis infection. The mixture probabilities (*p*_γ[*j*]_ and *p*_*u*[*k*]_) are to prevent temporal (structured γ_*i*[*j*]_ and unstructured δ_*i*[*j*]_) and spatial (structured *u*_*i*[*k*]_ and unstructured *v*_*i*[*k*]_) over-smoothing over large discontinuities, respectively.

The sample size $n_{i}=\left\{n_{i}^{*},n_{i}^{0}\right\}$ is a combination of observed samples $n_{i}^{*}$ and missing samples $n_{i}^{0}$. Since no information is available about the missingness of the observations, they were assumed to be missing at random (MAR), and a Bayesian imputation model was imposed for their prediction. The next stage of the model was the *imputation model*, which is to impute the missing samples as Poisson random variables with the total cattle counts *m*_*i*_ as offset, structured as


$$\begin{array}{l} f\left(n_{i}^{*},n_{i}^{0},m_{i}|\tau_{n\left(•\right)},\alpha \right)\sim Pois\left({\text{λ}}_{i}m_{i}\right)\\ \;\log{\text{λ}}_{i}\sim N\left(\alpha_{0}+z_{i\left[k\right]},\tau_{\text{λ}}\right) \end{array}$$

Here, we use the average spatial process *z*_*i*[*k*]_ of all the temporal steps centered by the intercept term α_0_ to borrow strength between neighboring observations.

In the Bayesian context, the next stage in modeling is the functional forms of the process layers, γ_*i*[*j*]_, δ_*i*[*j*]_, *v*_*i*[*k*]_, *u*_*i*[*k*]_, *U*_*i*[*k*]_. Based on different functional forms for the space, time, and space-time variables, three models were built. Model-1 includes a linear predictor that is decomposed into global spatial and temporal variations; that is


$$\begin{array}{l} \eta_{i}=\beta_{0}+p_{\gamma \left[j\right]}\gamma_{i\left[j\right]}+\left(1-p_{\gamma \left[j\right]}\right)\delta_{i\left[j\right]}+p_{u\left[k\right]}u_{i\left[k\right]}\\ \;+\left(1-p_{u\left[k\right]}\right)v_{i\left[k\right]} \end{array}$$

where, γ_*i*[*j*]_ and δ_*i*[*j*]_ are structured and unstructured temporal trends; and *u*_*i*[*k*]_ and *v*_*i*[*k*]_ are structured and unstructured spatial trends. For the structured temporal trends, a first-order random walk process was imposed such that γ_*i*[*j*]_~*N*(γ_*i*[*j*−1]_, τ_γ_) for *j* = 2, …, *J* and γ_*i*[1]_~*N*(0, τ_γ_). Here, the δ_*i*[*j*]_ and *v*_*i*[*k*]_ were modeled as exchangeable random intercepts, where δ_*i*[*j*]_~*N*(0, τ_γ_), *v*_*i*[*k*]_~*N*(0, τ_*v*_), for global temporal and spatial smoothing, respectively. For the structured spatial random effects *u*_*i*[*k*]_, a Gaussian Markov random field (GMRF) process was chosen for local smoothing. The precision matrix for the GMRF smoothing was based upon the spatial arrangements of the Missouri counties. The precision matrix has entries of −1 for counties that are neighbors, 0 for those that are not neighbors, and the number of neighboring counties as the diagonal elements. To reduce over-smoothing over large discontinuities, the random effects, temporal, and spatial terms were modeled as mixture random effects with mixture probabilities *p*_γ[*j*]_ and *p*_*u*[*k*]_, respectively.

In Model-2, a separate temporal smoothing function was introduced for each spatial entity, and a separate spatial smoothing function was introduced for each temporal unit; thus,


$$\begin{array}{l} \eta_{i}=\beta_{0}+p_{\gamma \left[j,k\right]}\gamma_{i\left[j,k\right]}+\left(1-p_{\gamma \left[j,k\right]}\right)\delta_{i\left[j,k\right]}+p_{u\left[j,k\right]}u_{i\left[j,k\right]}\\ \;+\left(1-p_{u\left[i,k\right]}\right)v_{i\left[j,k\right]} \end{array}$$

Model-2 assumes no space-time interaction effects but relaxes the assumption of universal spatial smoothing across all time points and temporal smoothing across all spatial entities.

Model-3 is an extension of Model-2 with additional space-time interaction effects. Here, a first-order dynamic CAR was specified, where *j* = 2, …, *J*, and *u*_*i*[*j, k*]_ = ρ_*i*[*k*]_*u*_*i*[*j*−1, *k*]_+*w*_*i*[*j, k*]_, where *u*_*i*[1, *k*]_ is an average CAR spatial process and *w*_*i*[*j, k*]_ are time-specific CAR processes. This is motivated by the class of dynamic models proposed by Gelfand, et al. (34). The temporal smoothing parameter ρ_*i*[*k*]_ spatially varies across the *k* spatial components. The mixture probabilities *p*_γ[*j, k*]_ and *p*_*u*[*j, k*]_ were modeled as random probabilities sampled from the Beta distribution; *p*_γ[*j, k*]_~β(1, 1), *p*_*u*[*j, k*]_~β(1, 1). Similarly, the common smoothing parameter was modeled as ρ_*i*[*k*]_~β(1, 1 ).

Finally, hyper-parameters were assigned to all the variance components, $\sigma_{\left\{•\right\}}^{2}$. For $\sigma_{\left\{•\right\}}^{2}=\tau_{\left\{•\right\}}^{-1}$, τ_{•}_~γ(0.5, 0.05) was assigned. The model was simulated using the *R2jags* R package (35) with two chains for 10,000 iterations each. The first 5,000 iterations were discarded, and the remaining 5,000 iterations for each chain were used for making Bayesian inference. Autocorrelation trace plots of the MCMC samples were checked for convergence, and the discrepancy function below was used as the goodness-of-fit statistic to check the adequacy of the models by comparing the observed and predicted anaplasmosis counts.


$$\begin{array}{l} \chi_{i}^{2}=\frac{{\left\{y_{i}-\text{𝔼}\left(y_{i}|\ldots \right)\right\}}^{2}}{V\left(y_{i}|\ldots \right)} \end{array}$$

This statistic is computed for the observed and predicted anaplasmosis counts and then compared for each MCMC iteration. The binomial expectation for the observed *E*(*y*_*i*_|…) = *n*_*i*_*p*_*i*_ matches that of the predicted. Similarly, the binomial variance of the observed anaplasmosis counts, *V*(*y*_*i*_|…) = (1−*p*)*E*(*y*_*i*_|…), equals that of the predicted counts. These two values are compared by calculating the Bayesian exceedance probabilities $\mathrm{Pr}\left(\overset{2}{\underset{i,obs}{\chi }}\geq \chi_{i,pred}^{2}\right)$. If there is no significant difference between the observed and predicted counts, the Bayesian *p*-values should be approximately 0.5. Bayesian *p*-values of < 0.1 or >0.9 are considered extreme (36).

A simpler explanation of the main differences between models 1, 2, and 3 and the reason for including a space-time interaction effect is provided in Supplementary File 1.

## 3 Results

### 3.1 Descriptive statistics

**Figure 1:**
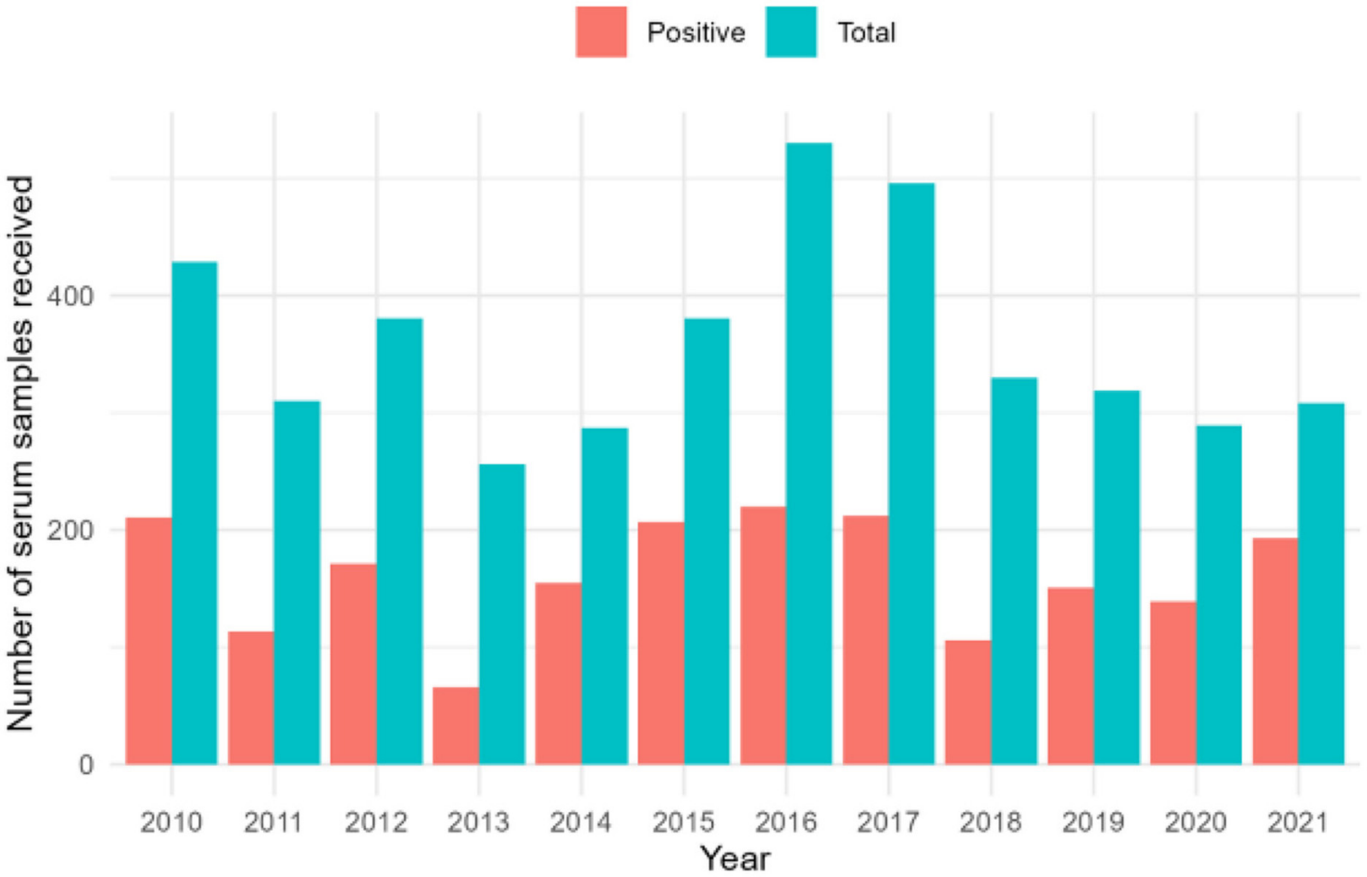
Annual counts of bovine serum samples submitted for *Anaplasma marginale* cELISA at the University of Missouri Veterinary Medical Diagnostic Laboratory, 2010–2021.

**Figure 2:**
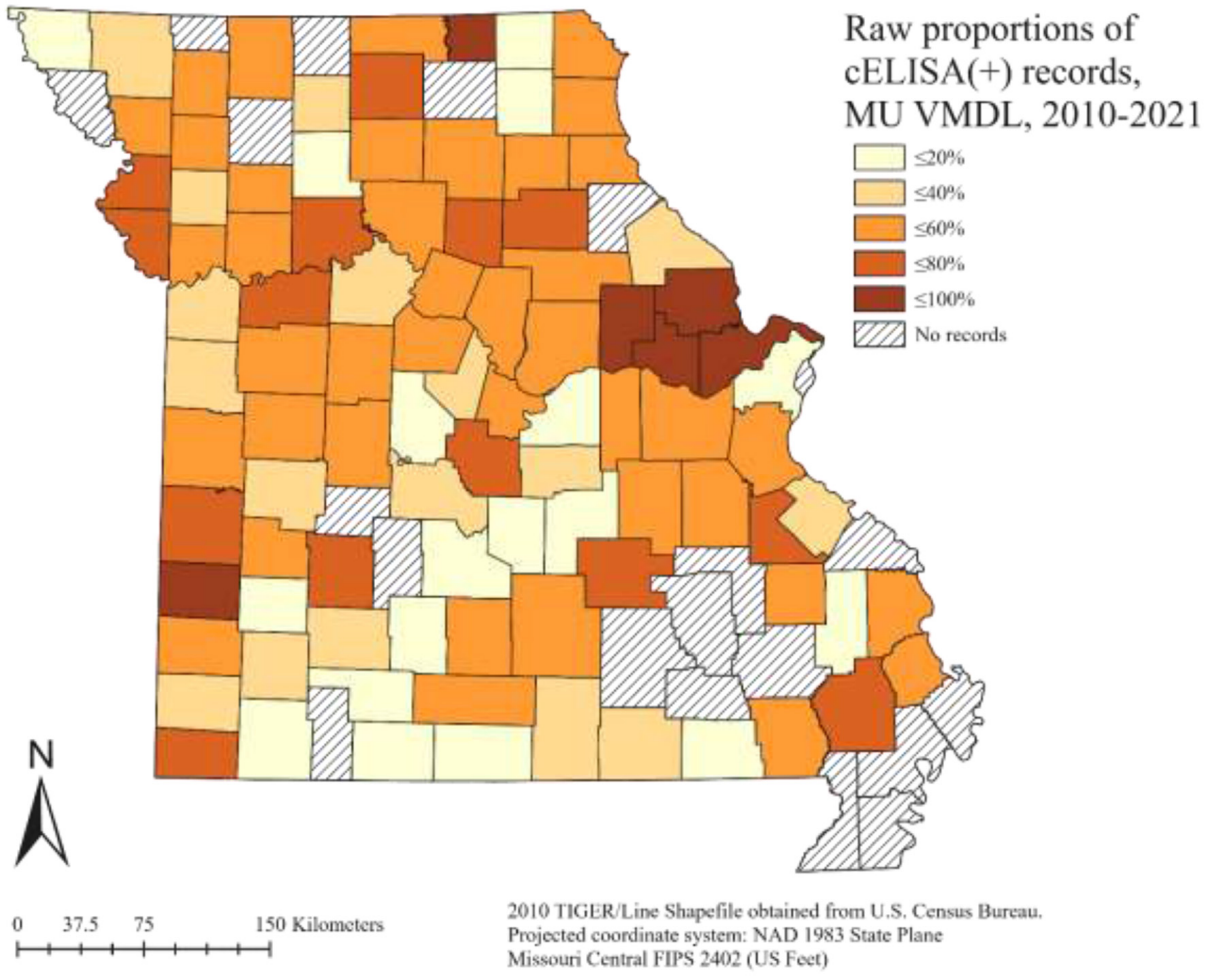
Raw proportions of bovine *Anaplasma* cELISA-positive records (normalized by all cELISA test records) submitted to the University of Missouri Veterinary Medical Diagnostic Laboratory, 2010–2021, summarized by county.

The final dataset used for analysis in this study (4,316 records) included 1,944 samples that tested positive for *A. marginale* by cELISA. The VMDL received a mean of 360 samples/year, with a mean of 162 positives/year (Figure 1). The largest number of both total and positive samples was received in 2016 (total = 530, positives = 220), and the smallest in 2013 (total = 256, positives = 66). Over the 12-year study period, the VMDL received a mean of 37.5 samples/county, with a mean of 16.9 positives/county. As expected, no obvious trends were apparent when crude counts were plotted at the county level (Figure 2).

### 3.2 Model estimations

**Figure 3:**
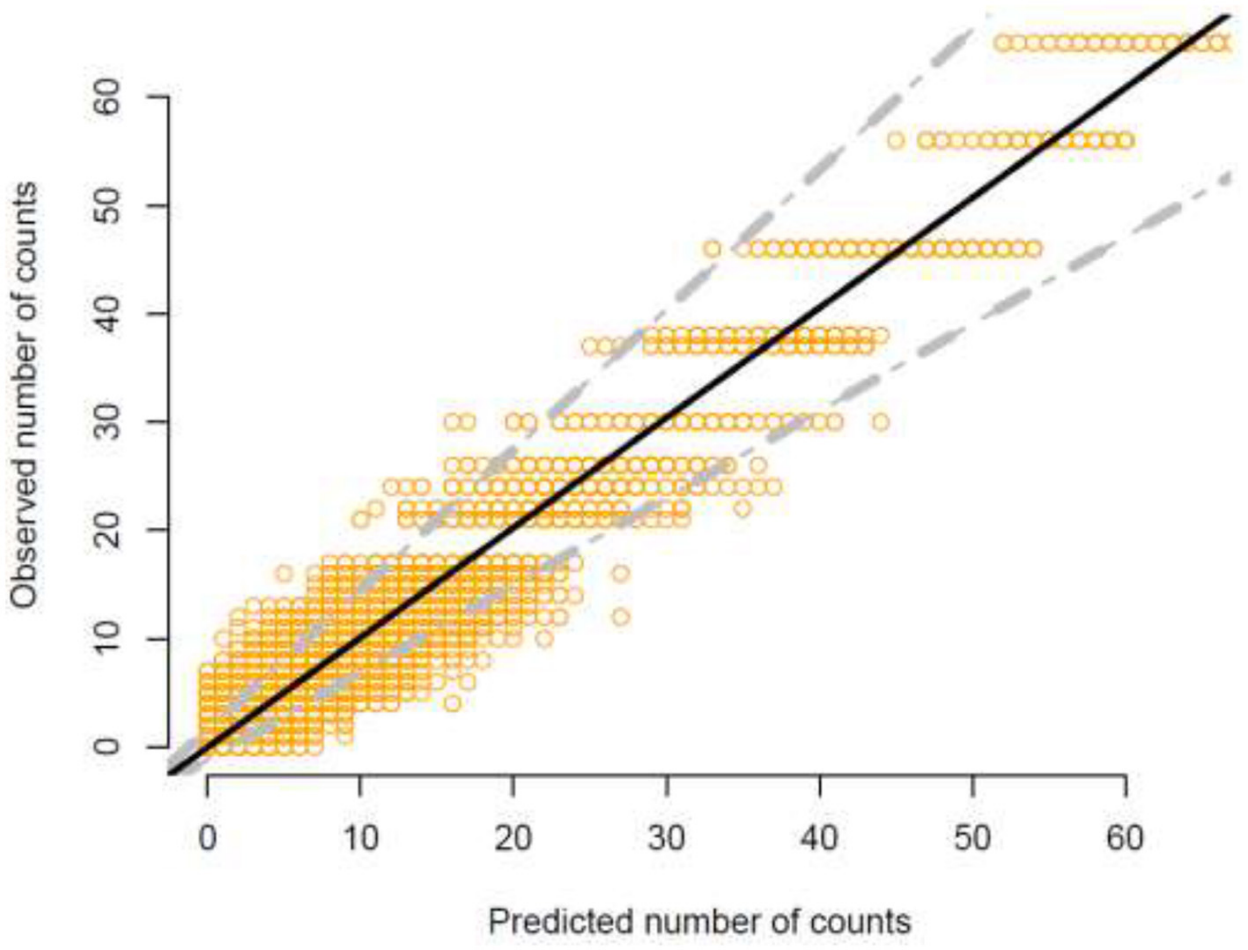
Predictive accuracy of the missing data imputation sub-model. Strong predictive accuracy is visible, with a coefficient of determination close to 1.

The dataset used for modeling contained approximately 58% missing records in the binomial sampling sizes, necessitating the inclusion of missing data imputation sub-models. These sub-models were identical across Models 1–3. As illustrated in Figure 3, the missing data imputation sub-model demonstrated strong predictive accuracy, with a coefficient of determination close to 1 (R^2^ ≈ 1). This imputation approach allowed for the use of the full dataset without excluding missing records.

Table 2 presents the DIC values used for model comparison. A lower DIC value indicates a better model. Model-3 outperforms the baseline Model-1 as well as Model-2. This outcome is anticipated since Model-1 applies global temporal and spatial smoothing functions, which is overly simplistic and impractical, particularly when the data is unstructured. Model-2 shares structural similarities with Model-3, as it applies separate spatial (temporal) smoothing to each temporal (spatial) unit. However, Model-3 surpasses Model-2 due to the inclusion of space-time interaction effects, which are essential for capturing the spatiotemporal dynamics of biological processes influencing anaplasmosis epidemiology. Consequently, all subsequent adequacy checks and interpretations were based on Model-3 alone.

**Table 2:** Evaluation parameters of the three Bayesian models constructed in the study.

**Model**	**Deviance information criterion**
Model-1	7,606.694
Model-2	5,223.553
Model-3	5,171.446

Lower Deviance Information Criterion (DIC) values indicate better model performance.

Model-3 includes a single fixed effect, β_0_ = −0.4, with a logit-inverse value of *logit inverse*(β_0_) = 0.40. This value represents the average risk of anaplasmosis infection, accounting for unknown exposure effects captured through space-time interaction effects.

**Figure 4:**
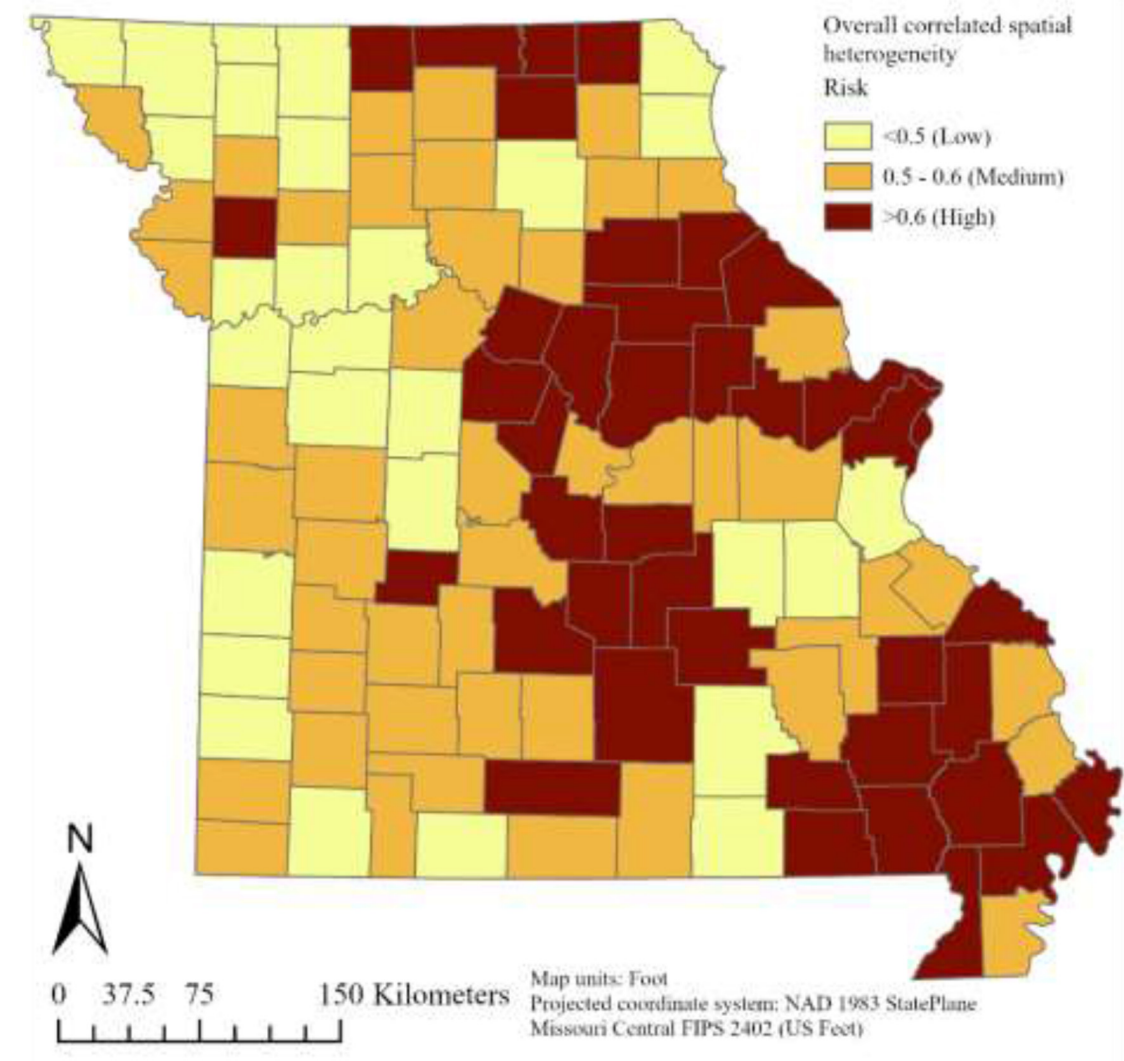
County-specific Bayesian posterior risk estimates of overall structured spatial heterogeneity.

**Figure 5:**
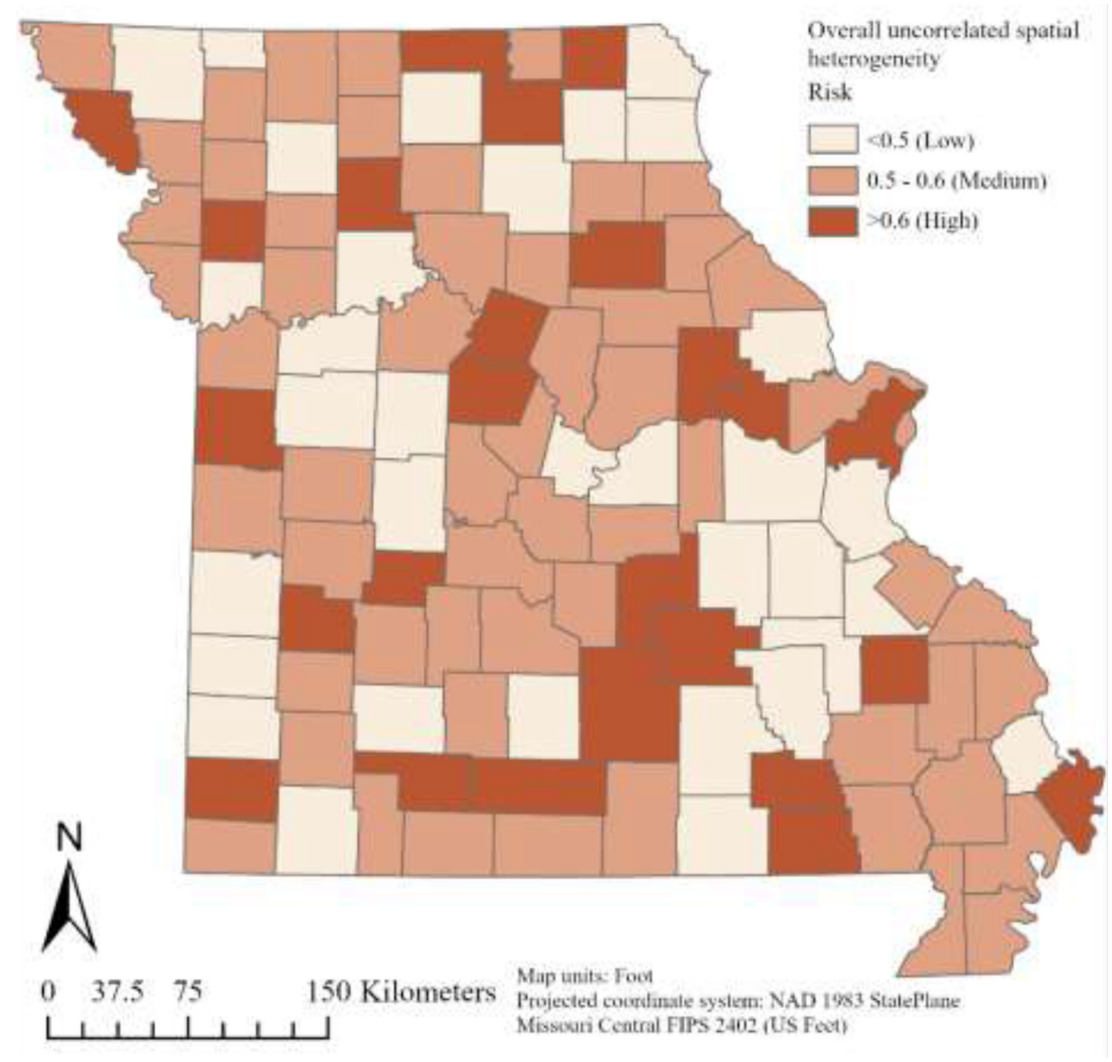
County-specific Bayesian posterior risk estimates of overall unstructured spatial heterogeneity.

**Figure 6:**
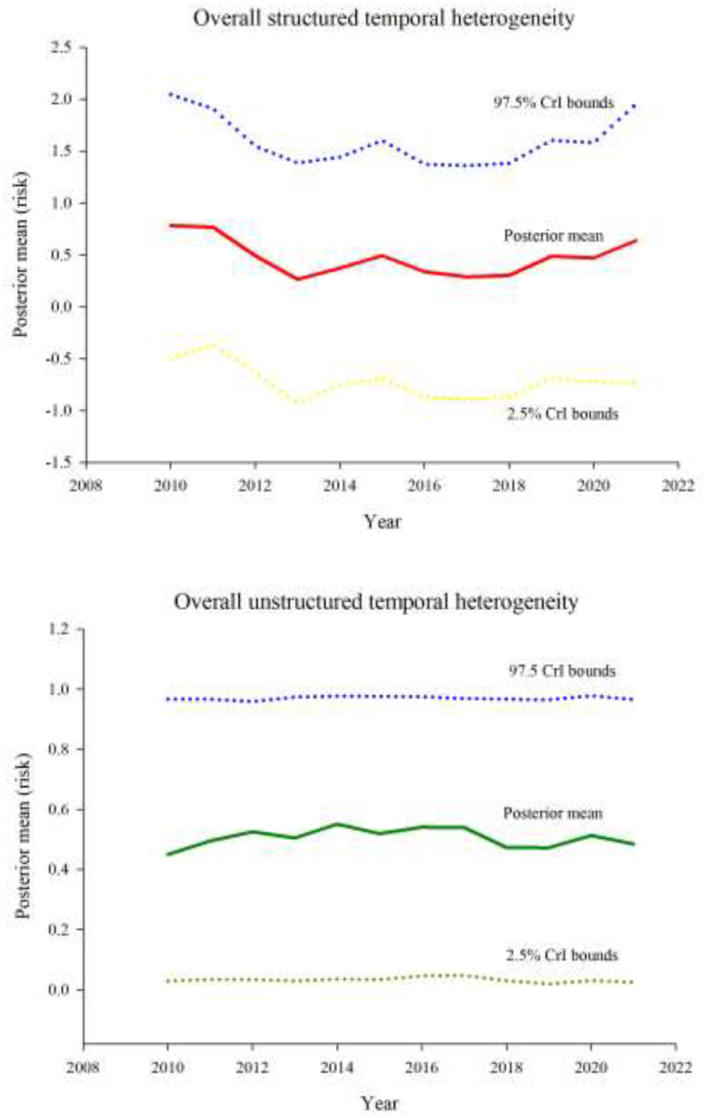
Purely temporal trends in Bayesian posterior mean risk along with 2.5% and 97.5% credible interval bounds of bovine anaplasmosis seropositivity risk in Missouri, 2010–2021, evaluated with structured **(top)** and unstructured **(bottom)** random effects.

**Figure 7:**
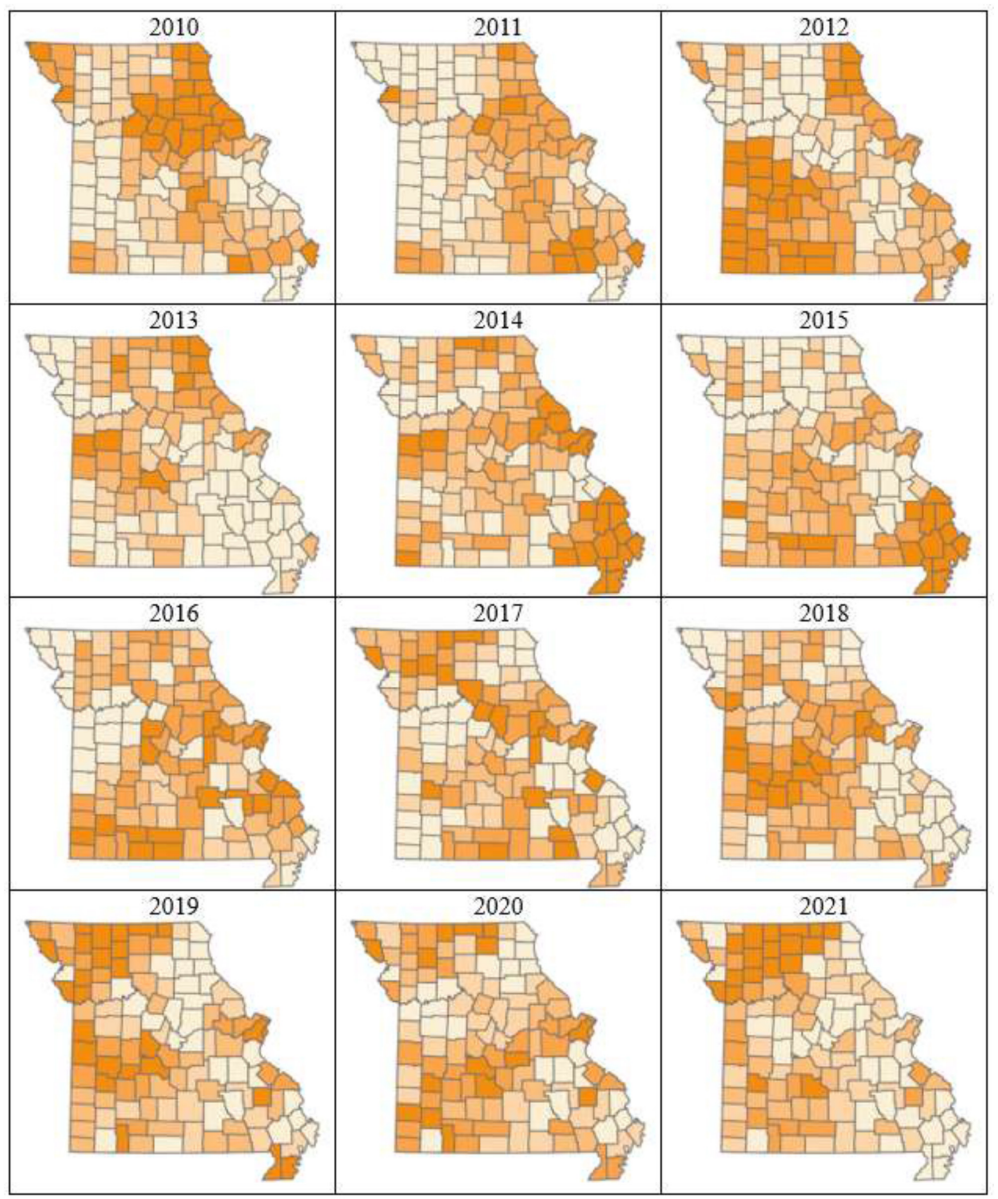
County- and year-specific Bayesian posterior risk estimates of structured spatial heterogeneity. Contiguous counties with similar colors indicate presence of same or similar underlying spatial processes that affect bovine anaplasmosis seroprevalence. Varying trends of higher and lower disease risk appearing to expand and contract over the years is observed.

**Figure 8:**
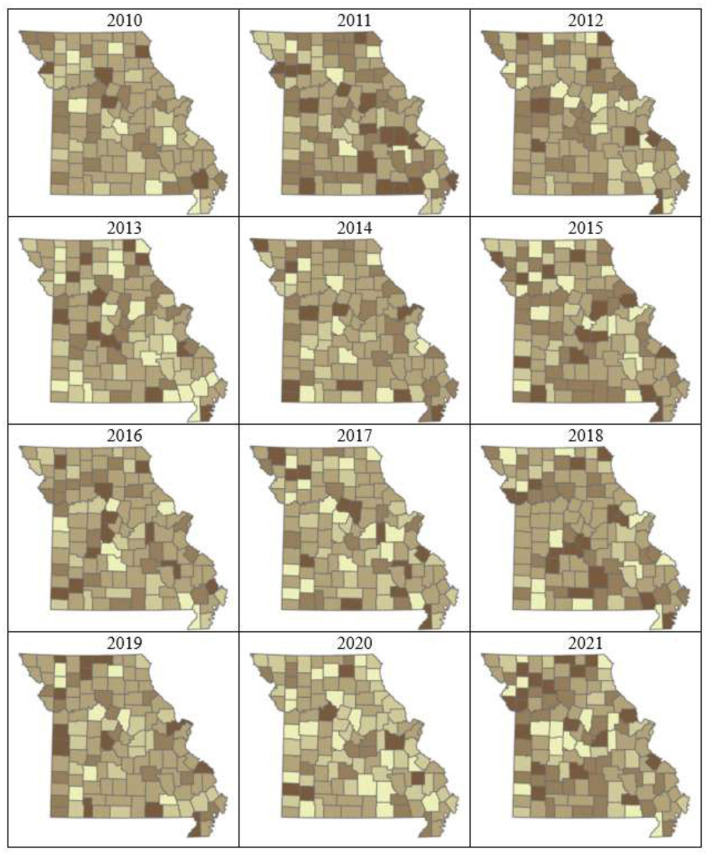
County- and year-specific Bayesian posterior risk estimates of spatially unstructured heterogeneity. Darker colors indicate anaplasmosis prevalence in those counties is driven by unique factors, rather than broader spatial processes operating at larger geographic scales spanning multiple contiguous counties. No trend is observed.

Plots of risk for the individual random effect components, derived from the logit inverse of posterior Bayesian estimates in Model-3, revealed a non-uniform spatiotemporal distribution of bovine anaplasmosis seroprevalence risk across Missouri counties. The overall spatial trend, assessed through the spatially structured and unstructured random effect components of Model-3, is presented in Figures 4, 5. Likewise, the overall temporal trend, evaluated using the temporally structured and unstructured random effects, is shown in Figure 6. Finally, the spatiotemporal risk is depicted through the structured spatiotemporal random effect (Figure 7) and the spatiotemporally unstructured random effect term (Figure 8).

**Figure 9:**
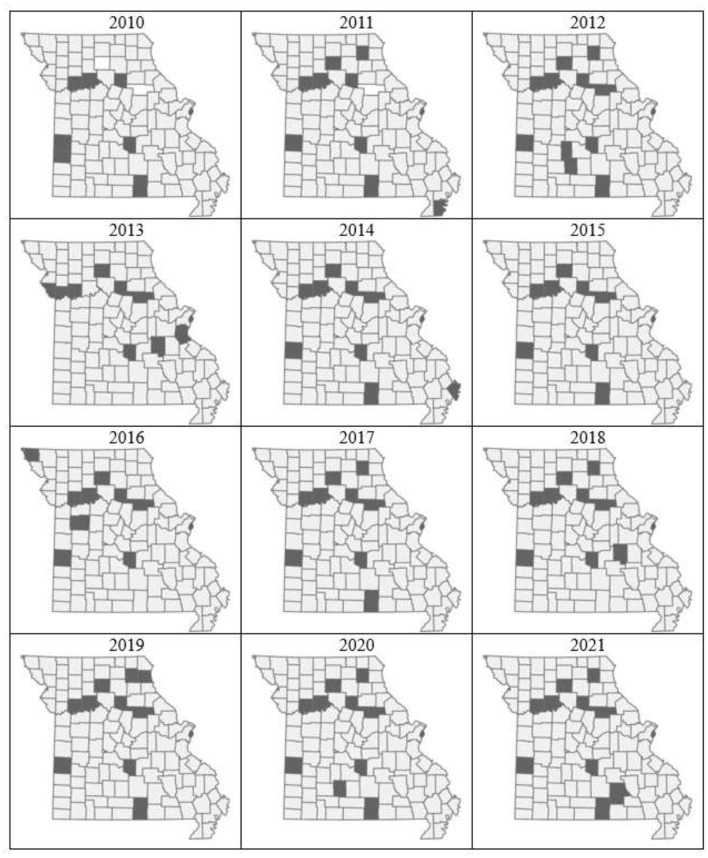
Bayesian *P-*values of exceedance probabilities comparing county- and year-specific observed and predicted bovine anaplasmosis seropositivity counts. Counties with extreme *p*-values < 0.1 and >0.9, indicating poor predictive performance, are highlighted in dark gray. For most years, the model performance is poor in a consistent set of Missouri counties.

Spatiotemporal plots of Bayesian *p*-values for exceedance probability (Figure 9) indicate that while Model-3 performs well for most counties in Missouri, there is a consistent set of counties where the model underperforms.

## 4 Discussion

Bovine anaplasmosis is endemic in Missouri, with an estimated statewide seroprevalence of 46.5%. (37). This study analyzed the current spatial and spatiotemporal patterns in the disease's distribution across the state using retrospective diagnostic data routinely recorded in a laboratory information management system (LIMS), often a source of information for making epidemiological and disease management decisions (38, 39). The Bayesian models considered in this study were data-driven, and the better performing model (Model-3) utilizes the CAR autocorrelation, which is effective in borrowing information from neighboring space-time units (27, 40–42). A strength of the models in this study is their ability to accommodate missing counts as well as missing sampling sizes, which alleviates the missing data problem often encountered in passive surveillance datasets. Such model structure is widely applied in site-occupancy models for accommodating local population uncertainties (33), and could be useful in situations similar to ours wherein LIMS data are assessed. The risk calculated in this study, i.e., the inverse logit of Bayesian posterior means for the purely spatial, purely temporal, and spatiotemporal random effects fitted in Model-3 reveal the spatial, temporal, and spatiotemporal trends for bovine anaplasmosis seropositivity over the 12-year study period.

The overall structured spatial heterogeneity (Figure 4), when classified in three categories (low, medium, and high risk), shows clusters of high-risk counties for bovine anaplasmosis seropositivity along the eastern half of the state, in the north, central and southeast, while the medium and low risk counties are found throughout, and are also found in contiguous groups of counties. This suggests that the underlying spatial processes that drive such clustering are likely to be similar or potentially arise from the same source, i.e., environmental, disease management practices by producers and veterinarians, or other similar factors. The overall unstructured spatial heterogeneity on the other hand (Figure 5) does not reveal any clustering, suggesting that there is no consistency in the effect of any local (or county-level) factors that play a major role in the spatial dynamics of bovine anaplasmosis seroprevalence in Missouri. The temporally structured and unstructured heterogeneity that accounted for the temporal effect in the retrospective dataset are presented in Figure 6, revealing that the risk of observing bovine anaplasmosis seroprevalence in Missouri has changed over the study period, with some years recording higher risk than others. While the trend is non-linear, it appears the disease burden is more or less stable over the years in Missouri, with some noticeable interannual variability.

Choropleth plots showing posterior risk of structured spatiotemporal heterogeneity? (Figure 7) indicates that the variations in presence of seropositive cases across Missouri counties are not independent but are spatially correlated, and shows a varying trend, with the disease risk appearing to expand and contract over the years. Disease risk increased significantly between 2010 and 2012, peaked around 2016, and again in 2019. The northeastern and southeastern regions consistently show higher risk throughout the years. After 2019, a decline in risk is noticeable; however, this trend could be likely due to less information available to the model that could be derived from the dataset for the latter years. Additionally, values outside these clusters change abruptly, with high and low-value counties scattered throughout the study area, sometimes in proximity. This pattern indicates that while the model successfully identifies regions potentially with similar characteristics affecting presence of seropositive cases, other unknown or unexamined factors may also contribute to the spatial distribution of the disease risk.

The risk maps of structured spatiotemporal heterogeneity further reveal that certain counties remain high-risk over multiple years, indicating persistent high-risk areas. These regions likely have stable environmental factors conducive to disease transmission, such as tick-favorable habitats or frequent livestock movement. Conversely, some counties consistently appear low-risk, representing stable low-risk areas where environmental or management conditions may naturally limit anaplasmosis transmission. Additionally, several counties exhibit fluctuating risk levels over time. Some areas increase in risk in later years, while others decline in risk. These shifts may result from interannual variations such as El Niño Southern Oscillation effects, seasonal variations, land use changes, or changes in disease management practices. Furthermore, emerging high-risk zones are evident, as some counties that were previously lower-risk become high-risk over time. This expansion of high-risk areas may be driven by factors such as climate change, shifts in livestock distribution, or increasing tick populations.

In contrast, the unstructured or spatially unstructured heterogeneity (Figure 8) captures variations in anaplasmosis prevalence that are independent across counties, without spatial correlation to neighboring areas. Higher values suggest that anaplasmosis risk in those counties is driven by unique factors rather than broader spatial processes that operate at larger geographic scales spanning multiple contiguous counties. While values vary widely across the study area, no distinct clusters emerge. This indicates that although global trends exist, local deviations also occur, where specific local level factors may influence individual counties differently. Finally, the spatiotemporal plot of Bayesian *p*-values for exceedance probability (Figure 9) indicates that while Model-3 performs well for a vast majority of the counties in Missouri, there is a consistent set of counties in Missouri where the model underperforms. One of the reasons for this underperformance could be related to fewer submissions from these counties to the VMDL, which are not adequately addressed by the missing data imputation method used. Additionally, this is also potentially due to the existence of unique factors that drive seropositivity risk in these counties, which are not adequately captured by the random effect terms included in Model-3. This can be alleviated by including biological (environmental, physical), and disease management-related variables that may capture the underlying spatiotemporal process more efficiently. Although our models did not directly evaluate the effects of environmental or management covariates, the spatial and temporal patterns observed in Missouri suggest that factors such as the distribution of wooded vs. open pastureland, regional variation in climate and vector habitat suitability, and differences in herd management practices (e.g., grazing intensity, herd movement, and vector control) may help explain variation in anaplasmosis risk. Incorporating these data into future analyses would allow for more precise assessments of how such factors shape disease dynamics and could guide the development of region-specific management strategies to reduce the burden of bovine anaplasmosis.

## 5 Conclusions

Clusters of high-risk counties scattered throughout the state (Figure 4) suggest the presence of similar underlying processes that operate across local and/or regional scales and which are not captured in the present model. Possible explanatory variables include local variations in microhabitat and host abundance, which can influence populations of vector ticks, such as soil characteristics (43), encroachment by woody plants such as invasive red cedar (44), and variations in population density of white-tailed deer (45). Local differences in cattle population and movement are also likely to be involved. The lack of any statewide trend in plots of spatially unstructured heterogeneity (Figures 5, 8) further illustrates the importance of local risk factors to anaplasmosis epidemiology in Missouri.

While the overall risk of bovine anaplasmosis seropositivity in Missouri appears relatively steady during the 12-year study period (Figure 6), some fluctuation is nonetheless apparent. While some of this fluctuation may be attributable to random “noise” in the available data, other potential explanatory variables not captured in the present model include temporal fluctuations in weather patterns, which in turn can influence cattle population dynamics through their effects on market pressures. A specific example is the marked reduction in beef cow inventories prompted by drought in 2011–2013, which reached its nadir in 2014 (46, 47). This was eventually followed by restocking of herds, often with replacement heifers shipped in from out-of-state, which increases the opportunities for comingling of naïve and carrier cattle (48). Temporal fluctuations in weather patterns are also likely to affect tick populations and host-seeking behaviors (49, 50).

Plots of county- and year-specific spatially structured heterogeneity (Figure 7) reveal four distinct situations: (1) counties that are consistently higher-risk, (2) counties that are consistently lower-risk, (3) counties that transition from lower to higher risk, and (4) counties that transition from higher to lower risk. Illustrated differently, if the faceted plot in Figure 7 were animated as a time series, the color gradient of certain counties would become progressively darker (increasing risk) or lighter (decreasing risk), sometimes multiple times during the study period. This is uncharacteristic of biological diffusion, i.e., “natural” spread of disease cases over space and time (51) and instead suggests introduction of cases via cattle movements, similar to the manner in which human diseases can appear in new locations via travel (52). Cattle movement, generally through markets, is strongly suspected to be an important source of variation in anaplasmosis risk around Missouri.

Finally, spatiotemporal plots of Bayesian *p*-values for exceedance probability (Figure 9) indicate 13 counties where the model performs poorly. Some are likely attributable to insufficient submissions from these counties to the VMDL; the independent city of St. Louis, which, as expected for beef cattle, has no submissions during the study period, is perhaps the best example. Nonetheless, several other counties (such as Dunklin and New Madrid, in the “bootheel” of southeastern Missouri) are also without submissions during the study period and are not flagged as underperforming via exceedance probabilities. Further work is required to understand why Model-3 performs poorly in some counties. One potential way to improve the model performance is by including fixed effect terms that may have further explanatory potential, such as those in the categories of environmental, herd management, and tick distribution. The availability of such data at the scale of present analysis and their suitable spatial transformation may however be limited.

## Data Availability

The data analyzed in this study is subject to the following licenses/restrictions: Data available for reasonable research use from the MU Veterinary Medical Diagnostic Laboratory. Requests to access these datasets should be directed to raghavanrk@missouri.edu.

## References

[1] Railey AF Marsh TL. Economic benefits of diagnostic testing in Livestock: anaplasmosis in cattle. Front Vet Sci. (2021) 8:626420. doi: 10.3389/fvets.2021.626420 34414221 PMC8369028

[2] Salinas-Estrella E Amaro-Estrada I Cobaxin-Cárdenas ME Preciado de la Torre JF Rodríguez SD. Bovine Anaplasmosis: Will there ever be an almighty effective vaccine? Front Vet Sci. (2022) 9:946545. doi: 10.3389/fvets.2022.946545 36277070 PMC9581321

[3] Curtis AK Kleinhenz MD Anantatat T Martin MS Magnin GC Coetzee JF . Failure to eliminate persistent *Anaplasma marginale* infection from cattle using labeled doses of chlortetracycline and oxytetracycline antimicrobials. Vet Sci. (2021) 20:8. doi: 10.3390/vetsci8110283 34822656 PMC8621018

[4] Toillion AR Reppert EJ Amachawadi RG Olson KC Coetzee JF Kang Q . Effect of protracted free-choice chlortetracycline-medicated mineral for anaplasmosis control on Escherichia coli chlortetracycline resistance profile from pastured beef cattle. Microorganisms. (2021) 9:2495. doi: 10.3390/microorganisms9122495 34946097 PMC8704331

[5] Reinbold JB Coetzee JF Hollis LC Nickell JS Riegel CM Christopher JA . Comparison of iatrogenic transmission of *Anaplasma marginale* in Holstein steers via needle and needle-free injection techniques. Am J Vet Res. (2010) 71:1178–88. doi: 10.2460/ajvr.71.10.1178 20919904 PMC8284935

[6] Scoles GA Miller JA Foil LD. Comparison of the efficiency of biological transmission of *Anaplasma marginale* (Rickettsiales: Anaplasmataceae) by *Dermacentor andersoni* Stiles (Acari: Ixodidae) with mechanical transmission by the horse fly, *Tabanus fuscicostatus* Hine (Diptera: Muscidae). J Med Entomol. (2008) 45:109–14. doi: 10.1093/jmedent/45.1.109 18283950

[7] Scoles GA Broce AB Lysyk TJ Palmer GH. Relative efficiency of biological transmission of *Anaplasma marginale* (Rickettsiales: Anaplasmataceae) by *Dermacentor andersoni* (Acari: Ixodidae) compared with mechanical transmission by *Stomoxys calcitrans* (Diptera: Muscidae). J Med Entomol. (2005) 42:668–75. doi: 10.1093/jmedent/42.4.668 16119558

[8] Jones AL Berghaus RD Kalatari AA Credille B Naikare HK Heins B . Seroprevalence and molecular detection of *Anaplasma marginale* infected beef herds in Georgia, USA. Bovine Pract. (2022) 56:70–8. doi: 10.21423/bovine-vol56no2p70-78

[9] Johnson-Walker YJ Steckler T Beever J Myint MS Rijwani S Beever E. Epidemiology and economic impact of anaplasmosis in southern Illinois beef cattle. Int Symp Vet Epidemiol Econ. (2018) 41.

[10] Okafor CC Collins SL Daniel JA Harvey B Sun X Coetzee JF . Factors associated with seroprevalence of anaplasma marginale in Kentucky cattle. Vet Parasitol Reg Stud Reports. (2018) 13:212–9. doi: 10.1016/j.vprsr.2018.07.003 31014877

[11] Okafor CC Collins SL Daniel JA Harvey B Coetzee JF Whitlock BK. Factors associated with seroprevalence of bovine anaplasmosis in Texas. Vet Parasitol Reg Stud Reports. (2018) 14:32–40. doi: 10.1016/j.vprsr.2018.08.004 31014734

[12] Okafor CC Collins SL Daniel JA Coetzee JF Whitlock BK. Seroprevalence of bovine anaplasmosis in Georgia. Vet Parasitol Reg Stud Reports. (2019) 15:100258. doi: 10.1016/j.vprsr.2018.100258 30929935

[13] Okafor CC Collins SL Daniel JA Coetzee JF Whitlock BK. Factors associated with seroprevalence of bovine anaplasmosis in Mississippi, USA. Vet Parasitol Reg Stud Reports. (2019) 17:100301. doi: 10.1016/j.vprsr.2019.100301 31303216

[14] Hanzlicek GA Raghavan RK Ganta RR Anderson GA. Bayesian space-time patterns and climatic determinants of bovine anaplasmosis. PLoS ONE. (2016) 11:e0151924. doi: 10.1371/journal.pone.0151924 27003596 PMC4803217

[15] Spare MR Hanzlicek GA Wootten KL Anderson GA Thomson DU Sanderson MW . Bovine anaplasmosis herd prevalence and management practices as risk-factors associated with herd disease status. Vet Parasitol X. (2020) 3:100021. doi: 10.1016/j.vpoa.2019.100021 32904721 PMC7458371

[16] Binder AM Armstrong PA. Increase in reports of tick-borne rickettsial diseases in the United States. Am J Nurs. (2019) 119:20–1. doi: 10.1097/01.NAJ.0000569428.81917.6c 31232769 PMC7053124

[17] Boorgula GDY Peterson AT Foley DH Ganta RR Raghavan RK. Assessing the current and future potential geographic distribution of the American dog tick, Dermacentor variabilis (Say) (Acari: Ixodidae) in North America. PLoS ONE. (2020) 15:e0237191. doi: 10.1371/journal.pone.0237191 32776959 PMC7416948

[18] Raghavan RK Peterson AT Cobos ME Ganta R. Foley D. Current and future distribution of the Lone Star Tick, *Amblyomma americanum* (L) (Acari: Ixodidae) in North America. PLoS ONE. (2019) 14:e0209082. doi: 10.1371/journal.pone.0209082 30601855 PMC6314611

[19] Raghavan RK Koestel ZL Boorgula G Hroobi A Ganta R Harrington J . Unexpected winter questing activity of ticks in the Central Midwestern United States. PLoS ONE. (2021) 16:e0259769. doi: 10.1371/journal.pone.0259769 34762706 PMC8584693

[20] Dergousoff SJ Galloway TD Lindsay LR Curry PS Chilton NB. Range expansion of *Dermacentor variabilis* and *Dermacentor andersoni* (Acari: Ixodidae) near their northern distributional limits. J Med Entomol. (2013) 50:510–20. doi: 10.1603/ME12193 23802445

[21] Molaei G Eisen LM Price KJ Eisen RJ. Range expansion of native and invasive ticks: a looming public health threat. J Infect Dis 26. (2022) 226:370–3. doi: 10.1093/infdis/jiac249 35732174 PMC10860637

[22] Ierardi RA. A review of bovine anaplasmosis (Anaplasma marginale) with emphasis on epidemiology and diagnostic testing. J Vet Diagn Invest. (2025) doi: 10.1177/10406387251324180 40156087 PMC11955989

[23] Kollars TM Jr Oliver JH Jr Masters EJ Kollars PG Durden LA. Host utilization and seasonal occurrence of *Dermacentor species* (Acari:Ixodidae) in Missouri, USA. Exp Appl Acarol. (2000) 24:631–43. doi: 10.1023/A:1026566301325 11201355

[24] Raghavan RK Neises D Goodin DG Andresen DA Ganta RR. Bayesian spatio-temporal analysis and geospatial risk factors of human monocytic ehrlichiosis. PLoS ONE. (2014) 9:e100850. doi: 10.1371/journal.pone.0100850 24992684 PMC4081574

[25] Raghavan RK Goodin DG Neises D Anderson GA Ganta RR. Hierarchical Bayesian spatio-temporal analysis of climatic and socio-economic determinants of rocky mountain spotted fever. PLoS ONE. (2016) 11:e0150180. doi: 10.1371/journal.pone.0150180 26942604 PMC4778859

[26] Curtis AK Coetzee JF. Assessment of within-herd seroprevalence of Anaplasma marginale antibodies and associated decreased milk production in an Iowa dairy herd. Appl Animal Sci. (2021) 37:126–31. doi: 10.15232/aas.2020-02110

[27] Lawson AB. Bayesian Disease Mapping: Hierarchical Modeling in Spatial Epidemiology, 3rd ed. Boca Raton, FL: CRC Press (2018). p. 488. doi: 10.1201/9781351271769-1

[28] Enticott G Ward K Ashton A Brunton L Broughan J. Mapping the geography of disease: a comparison of epidemiologists‘ and field-level experts' disease maps. Appl Geogr. (2021) 126:102356. doi: 10.1016/j.apgeog.2020.102356

[29] MacNab YC. Bayesian disease mapping: past, present, and future. Spat Stat. (2022) 50:100593. doi: 10.1016/j.spasta.2022.100593 35075407 PMC8769562

[30] Marshall RJ. Mapping disease and mortality rates using empirical Bayes estimators. J R Stat Soc Ser C Appl Stat. (1991) 40:283–94. doi: 10.2307/2347593 12157989

[31] Kéry M Schaub M. Bayesian Population Analysis Using WinBUGS: A Hierarchical Perspective, 1st ed. Cambridge, MA: Academic Press (2011). p. 554. doi: 10.1016/B978-0-12-387020-9.00001-8

[32] Kéry M Royle JA. Chapter 2. Modeling population dynamics with count data. In:Kéry M Royle JA, editors. Applied Hierarchical Modeling in Ecology: Analysis of Distribution, Abundance and Species Richness in R and BUGS. Cambridge, MA: Academic Press. (2021). p. 65–156. doi: 10.1016/B978-0-12-809585-0.00002-8

[33] Royle JA. N-mixture models for estimating population size from spatially replicated counts. Biometrics. (2004) 60:108–15. doi: 10.1111/j.0006-341X.2004.00142.x 15032780

[34] Gelfand AE Banerjee S Gamerman D. Spatial process modelling for univariate and multivariate dynamic spatial data. Environmetrics. (2005) 16:465–79. doi: 10.1002/env.715

[35] R2jags: Using R to Run ‘JAGS'. Version 0.8-9. 2025. Available online at: https://cran.r-project.org/web/packages/R2jags/index.html (Accessed May 5, 2025).

[36] Tzala E Best N. Bayesian latent variable modelling of multivariate spatio-temporal variation in cancer mortality. Stat Methods Med Res. (2008) 17:97–118. doi: 10.1177/0962280207081243 17855747

[37] Ierardi RA Odemuyiwa SO Schultz L Shen Z Zhang M Zhang S . Serologic and molecular prevalence of Anaplasma marginale in Missouri beef herds. Am J Vet Res. (2025) 86:1–10. doi: 10.2460/ajvr.25.03.0096 40683302

[38] Sandlin CS Johnson RC Swaim L Ashley DL. Laboratory information management system for emergency response: validation and quality assurance of analytical methodologies. SLAS Technol. (2009) 14:126–32. doi: 10.1016/j.jala.2009.02.001

[39] Colangeli P De Massis F Cito F Mercante MT Ricci L. Laboratory information management systems: role in veterinary activities. In:Management Association IR, editor. Public Health and Welfare: Concepts, Methodologies, Tools, and Applications. Palmdale, PA: IGI Global (2017). p. 313–26. doi: 10.4018/978-1-5225-1674-3.ch015

[40] Knorr-Held L Besag J. Modelling risk from a disease in time and space. Stat Med. (1998) 17:2045–60 doi: 10.1002/(SICI)1097-0258(19980930)17:18&lt;2045::AID-SIM943&gt;3.0.CO;2-P 9789913

[41] Bernardinelli L Clayton D Pascutto C Montomoli C Ghislandi M Songini M. Bayesian analysis of space—time variation in disease risk. Stat Med. (1995) 14:2433–43. doi: 10.1002/sim.4780142112 8711279

[42] Banerjee S. High-dimensional bayesian geostatistics. Bayesian Anal. (2017) 12:583–614. doi: 10.1214/17-BA1056R 29391920 PMC5790125

[43] Rochlin I Egizi A Narvaez Z Bonilla DL Gallagher M Williams GM . Microhabitat modeling of the invasive Asian longhorned tick (Haemaphysalis longicornis) in New Jersey, USA. Ticks Tick Borne Dis. (2023) 14:102126. doi: 10.1016/j.ttbdis.2023.102126 36682197

[44] Noden BH Tanner EP Polo JA Fuhlendorf SD. Invasive woody plants as foci of tick-borne pathogens: eastern redcedar in the southern Great Plains. J Vector Ecol. (2021) 46:12–18, 7. doi: 10.52707/1081-1710-46.1.12 35229576

[45] Patrick CD Hair JA. White-tailed deer utilization of three different habitats and its influence on lone star tick populations. J Parasitol. (1978) 64:1100–6. doi: 10.2307/3279735

[46] United States Department of Agriculture National Agricultural Statistics Service (USDA-NASS). Quick Stats (Cattle, Cows, Beef - Inventory, Total). Available online at: https://quickstats.nass.usda.gov/ (Accessed March 6, 2025).

[47] Hrozencik RA. Drought conditions and change in U.S. beef cattle herd size, 2000–2023 (2024). United States Department of Agriculture Economic Research Service. Source: USDA, Economic Research Service using data from the U.S. Drought Monitor and USDA, National Agricultural Statistics Service.

[48] Hairgrove TB Craig TM Budke CM Rodgers SJ Gill RJ. Seroprevalence of Anaplasma marginale in Texas cattle. Prev Vet Med. (2014) 116:188–92. doi: 10.1016/j.prevetmed.2014.05.008 24931130

[49] Jones CJ Kitron UD. Populations of *Ixodes scapularis* (Acari: Ixodidae) Are modulated by drought at a lyme disease focus in illinois. J Med Entomol. (2000) 37:408–15. doi: 10.1093/jmedent/37.3.408 15535585

[50] Nielebeck C Kim SH Pepe A Himes L Miller Z Zummo S . Climatic stress decreases tick survival but increases rate of host-seeking behavior. Ecosphere. (2023) 14:e4369. doi: 10.1002/ecs2.4369

[51] Hefley TJ Hooten MB Russell RE Walsh DP Powell JA. When mechanism matters: Bayesian forecasting using models of ecological diffusion. Ecol Lett. (2017) 20:640–50. doi: 10.1111/ele.12763 28371055

[52] Tao Y Hite JL Lafferty KD Earn DJD Bharti N. Transient disease dynamics across ecological scales. Theor Ecol. (2021) 14:625–40. doi: 10.1007/s12080-021-00514-w 34075317 PMC8156581

