# Supplementary material for: Bayesian spatiotemporal evaluation of bovine anaplasmosis seroprevalence in Missouri (2010–2021)
Source: Front Vet Sci. 2026 Jan 23;12:1658248. doi: 10.3389/fvets.2025.1658248 (PMC12875914; doi:10.3389/fvets.2025.1658248)
Supplement: Supplementary file 1 [file Table_1.docx]

**Supplementary File 1.**

We considered three hierarchical models of increasing complexity. Model 1 decomposes variation into global temporal and spatial effects, assuming that all locations share a common temporal trend and all years share a common spatial pattern. Model 2 relaxes this assumption by allowing each spatial unit to have its own temporal trend, and each temporal unit to have its own spatial pattern, though without explicit interaction terms. Model 3 extends this framework further by including explicit space–time interactions, so that temporal dynamics can differ across locations and evolve in a spatially structured way.

Accounting for space–time interactions is important because disease processes rarely follow uniform trends across all regions or years; instead, the timing and intensity of outbreaks often vary geographically. Ignoring such interactions risks over-smoothing or masking localized epidemics.

Model 2

(Separate temporal per county, separate spatial per year, no interactions)

Model 3

(Explicity space-time interactions, counts evolve dynamically)

Model 1

(Global temporal + spatial trends)
